# Reviewers in 2021

**DOI:** 10.1098/rspb.2022.0239

**Published:** 2022-03-30

**Authors:** Proceedings B Editorial Team

The Editors of *Proceedings B* would like to thank all reviewers who contributed their time by refereeing manuscripts submitted throughout 2021. From previous feedback, we know that most reviewers value recognition of their efforts by the journal and through services, such as Publons, which is integrated with *Proceedings B*.

To provide an opportunity for recognition, we list the names of all reviewers (unless they have opted out). This article is made permanent and citeable by its digital object identifier (DOI). We encourage you to quote your contribution in your applications for tenure and grants or other forms of research assessment. Where you have provided it, we have included your ORCID, so your contribution can be unambiguously assigned to you.

The team really does appreciate all the work our reviewers do on behalf of the journal, and we look forward to working with you again in 2022.

A. Almeida R https://orcid.org/0000-0002-5228-9091
Abelson E https://orcid.org/0000-0003-0627-1111
Abolins-Abols M
Abouheif E
Abourachid A
Abrego N https://orcid.org/0000-0001-6347-6127
Acevedo J
Aceves A
Adachi T
Adamec L
Adena S
Adler F
Adrián AD
Adrian F
Aeles J https://orcid.org/0000-0003-1514-4958
Aerts P https://orcid.org/0000-0002-6867-5421
Aglieri G
Agrawal A https://orcid.org/0000-0003-0095-1220
Agren J
Ågren JA https://orcid.org/0000-0003-3619-556X
Ahrens R
Akbari OS
Albano P https://orcid.org/0000-0001-9876-1024
Albery GF
Albini A
Albon S
Albright R
Alec W
Alex L
Alex T
Alibardi L
Allen M https://orcid.org/0000-0002-6632-4337
Altschul D https://orcid.org/0000-0001-7053-4209
Alvarez-Carretero S
Amador Kane S https://orcid.org/0000-0001-7532-7532
Amanda C
Amaral Carvalho ME https://orcid.org/0000-0002-7221-5380
Amato K
Amorim D
Amos M https://orcid.org/0000-0003-1680-5535
Amundsen P-A
Andersen K https://orcid.org/0000-0002-8478-3430
Anderson J https://orcid.org/0000-0003-2441-0728
Anderson R https://orcid.org/0000-0002-0558-7563
Anderson S https://orcid.org/0000-0003-3096-1120
Anderson T
Andrea C
Andrus F
Angelini C
Angerbjorn A
Angielczyk K
Anjalika N
Anneliis S-T
Anreiter I
Anthwal N https://orcid.org/0000-0002-4104-7839
Antia R
Aoi S https://orcid.org/0000-0001-9243-2641
Aparajitha R https://orcid.org/0000-0002-7200-1366
Araya-Ajoy Y
Arbetman M https://orcid.org/0000-0002-8382-2522
Arellano C https://orcid.org/0000-0001-9672-3497
Aristide L
Armstrong J
Armstrong T
Arnedo M https://orcid.org/0000-0003-1402-4727
Arnold P https://orcid.org/0000-0002-6158-7752
Arnold P https://orcid.org/0000-0002-6310-5876
Arrizabalaga H
Ashley S
Ashton G
Askew G https://orcid.org/0000-0003-1010-4439
Athanasiadis I https://orcid.org/0000-0003-2764-0078
Aubier T https://orcid.org/0000-0001-8543-5596
Aubin-Horth N https://orcid.org/0000-0002-9030-634X
Aubree J
Audzijonyte A
Auer T
Aulet L
Avgar T https://orcid.org/0000-0002-8764-6976
Aznar FJ
Babak K
Babik W https://orcid.org/0000-0002-1698-6615
Bachelot B https://orcid.org/0000-0003-3348-9757
Backus E
Baggio J
Baines C https://orcid.org/0000-0002-6918-3648
Bairlein F
Bajic D https://orcid.org/0000-0002-6716-7898
Baker C
Bakker T
Balakrishnan C https://orcid.org/0000-0002-0788-0659
Balas B
Balasubramaniam K
Baliga V https://orcid.org/0000-0002-9367-8974
Balling R
Ballinger M
Bangal P
Baniaga A
Banks M
Barabas G
Barata C
Barbaro K
Barber J
Barber-Meyer S
Barclay P https://orcid.org/0000-0002-7905-9069
Barghi N https://orcid.org/0000-0003-3700-0971
Barnett J https://orcid.org/0000-0001-9789-4132
Barrett B https://orcid.org/0000-0002-2725-2385
Barrett R
Barron-Ortiz C
Bartzke G
Baruffaldi L http://orcid.org/0000-0003-2933-6969
Barve S https://orcid.org/0000-0001-5840-8023
Baryshnikov G
Basak SM
Baskett M
Bastille-Rousseau G https://orcid.org/0000-0001-6799-639X
Batstone R
Bau H https://orcid.org/0000-0003-3815-5422
Bauch C https://orcid.org/0000-0001-6214-6601
Bauch C https://orcid.org/0000-0003-0218-5582
Bauke S
Bay R
Bayle P
Beaumont J
Bebbington K https://orcid.org/0000-0001-7714-9100
Beck O
Beck R https://orcid.org/0000-0002-7050-7072
Becker D https://orcid.org/0000-0003-4315-8628
Beckerman A
Beckman N
Becks L https://orcid.org/0000-0002-3885-5253
Bedny M
Bee M https://orcid.org/0000-0002-6770-9730
Beehner J
Belgrad B https://orcid.org/0000-0003-2032-2210
Bell G https://orcid.org/0000-0002-7280-6391
Bellés X https://orcid.org/0000-0002-1566-303X
Beltran R
Bemmels J https://orcid.org/0000-0001-9996-6996
Bender A https://orcid.org/0000-0001-9542-2975
Benestan L https://orcid.org/0000-0003-1638-9893
Benham P
Benitez-Vieyra S
Benoit J
Beran M https://orcid.org/0000-0002-2504-0338
Berg E https://orcid.org/0000-0002-9637-8109
Berg J
Berger D https://orcid.org/0000-0003-0196-6109
Bernal X https://orcid.org/0000-0001-6155-5980
Bernardi G https://orcid.org/0000-0002-8249-4678
Berner D
Bertamini M
Best A
Bestion E https://orcid.org/0000-0001-5622-7907
Beye M
Bhattacharyya A
Bianchi E
Biek R
Bierne N
Bingyi Y https://orcid.org/0000-0002-0811-8332
Birky W
Bischof R
Bishop P
Bishop T
Bittleston L https://orcid.org/0000-0003-4007-5405
Björklund M
Blagrove R
Blanco-Bercial L https://orcid.org/0000-0003-0658-7183
Blanke A https://orcid.org/0000-0003-4385-6039
Blatrix R https://orcid.org/0000-0003-1662-7791
Blatt M
Blumenthal E
Blumstein D https://orcid.org/0000-0001-5793-9244
Bodensteiner B https://orcid.org/0000-0001-6628-1923
Boeckle M https://orcid.org/0000-0002-0738-2764
Boëda E
Boehne A
Bollazzi M https://orcid.org/0000-0003-3361-5602
Bombin A
Bonachela J https://orcid.org/0000-0002-3316-8120
Bonapersona V
Bonduriansky R https://orcid.org/0000-0002-5786-6951
Bonnet T
Bonsell C
Boogert N https://orcid.org/0000-0002-1337-4365
Booksmythe I
Boonman A https://orcid.org/0000-0002-3686-1246
Boratynski Z https://orcid.org/0000-0003-4668-4922
Borchering R https://orcid.org/0000-0003-4309-2913
Borniger JC
Borodich F https://orcid.org/0000-0002-7935-0956
Borregaard M https://orcid.org/0000-0002-8146-8435
Borzacchiello G
Bose A
Boström Einarsson L
Botero C
Botting J
Bouchenak-Khelladi Y
Bousquet C https://orcid.org/0000-0003-1650-8004
Bove C https://orcid.org/0000-0003-0340-1371
Bowles AMC https://orcid.org/0000-0002-7487-3811
Bowman D
Bowman JL https://orcid.org/0000-0002-0816-6115
Boyaci H
Boyce M
Boyd B https://orcid.org/0000-0002-9837-3483
Boyko C
Bradley J
Brand C https://orcid.org/0000-0003-1285-2174
Brandell E https://orcid.org/0000-0002-2698-7013
Braziunas K https://orcid.org/0000-0001-5350-8463
Breen A https://orcid.org/0000-0002-2331-0920
Brennan A
Brennan P
Brent L https://orcid.org/0000-0002-1202-1939
Bressman N
Brett T https://orcid.org/0000-0002-0906-441X
Breusing C
Breves J
Brian L
Brian L
Briedis M
Brink K https://orcid.org/0000-0001-6630-1525
Brinkløv S
Brisson D
Brisson J https://orcid.org/0000-0001-7000-0709
Brocklehurst N https://orcid.org/0000-0003-2650-5480
Brodribb T http://orcid.org/0000-0002-4964-6107
Bromaghin J
Brommer J https://orcid.org/0000-0002-2435-2612
Brooks A
Brooks M
Brooks R
Brown A
Brown A https://orcid.org/0000-0001-7022-5342
Brown C https://orcid.org/0000-0001-6463-8677
Brown C https://orcid.org/0000-0002-0210-1820
Brown C https://orcid.org/0000-0002-6226-3864
Brown E
Brown M https://orcid.org/0000-0002-8887-3628
Brownell R
Bruner E
Brunner J
Brynne D
Bshary R https://orcid.org/0000-0001-7198-8472
Buchholz S http://orcid.org/0000-0003-2018-7266
Buchner O https://orcid.org/0000-0003-0733-6562
Buckling A
Budd G https://orcid.org/0000-0001-9007-4369
Buffalo V
Bugg T
Büllesbach J
Bulley A https://orcid.org/0000-0001-8014-0078
Burdon F
Burgess M https://orcid.org/0000-0002-3750-4347
Burgess S https://orcid.org/0000-0002-0348-3453
Burghardt L
Burin G
Burness G
Burns M
Burns S https://orcid.org/0000-0001-8695-3643
Burr D https://orcid.org/0000-0003-1541-8832
Burraco P
Burridge C https://orcid.org/0000-0002-8185-6091
Burton P
Burton R
Bury T
Butchart S
Butlin R
Button D
Byrne M https://orcid.org/0000-0002-8902-9808
C Rajagopal M https://orcid.org/0000-0001-7230-2946
C. Sean B
Cabral JS
Caetano D
Calovi D https://orcid.org/0000-0002-2452-9801
Cambon M
Camerlink I https://orcid.org/0000-0002-3427-2210
Camille K
Campanya-Llovet N
Campos D
Campos F https://orcid.org/0000-0001-9826-751X
Camus M https://orcid.org/0000-0003-0626-6865
Cañestro C
Cant M https://orcid.org/0000-0002-1530-3077
Cantalapiedra J https://orcid.org/0000-0003-0913-7735
Cantin N
Cantor M https://orcid.org/0000-0002-0019-5106
Cao Y https://orcid.org/0000-0002-1732-6491
Capaldi A
Cappellini E
Capps K
Capraro V https://orcid.org/0000-0002-0579-0166
Carazo P https://orcid.org/0000-0002-1525-6522
Carcaud J
Cardona T http://orcid.org/0000-0003-2076-4115
Cardoso G https://orcid.org/0000-0001-6258-1881
Careau V https://orcid.org/0000-0002-2826-7837
Carina E
Carlos B
Caro T https://orcid.org/0000-0001-6804-8519
Carrano M
Carrascal LM
Carrier D
Carriquí M https://orcid.org/0000-0002-0153-2602
Carroll T
Carter G
Carver S https://orcid.org/0000-0002-3579-7588
Casagrande S https://orcid.org/0000-0002-4264-8062
Casares F https://orcid.org/0000-0002-2181-3858
Cassin Sackett L
Cassis G
Catarina SL
Catchen J
Cathcart C
Catherine S
Cattadori I https://orcid.org/0000-0001-6618-316X
Cazelles K https://orcid.org/0000-0001-6619-9874
Chadès I
Chaine A
Chambers C
Chaminade T https://orcid.org/0000-0003-4952-1467
Chan C
Chang C-W https://orcid.org/0000-0002-9817-2956
Chang H
Charlie W
Chaumeil J
Chelo I
Chen A
Chen I-C
Chen Y
Chen Z http://orcid.org/0000-0003-0575-7013
Chenard C
Chevin L-M https://orcid.org/0000-0003-4188-4618
Chiarenza A https://orcid.org/0000-0001-5525-6730
Chierchia G https://orcid.org/0000-0002-5623-4573
Chiou K
Chipman A https://orcid.org/0000-0001-6696-840X
Chira A https://orcid.org/0000-0002-2964-7583
Chislock M
Chloe W
Choat J
Chown S
Chris L
Christie K https://orcid.org/0000-0001-8257-2106
Christy J
Christy N
Chu C-J
Chuard P http://orcid.org/0000-0001-5414-0242
Chudzinska M
Chung D
Cid Puey N
Civelek Z https://orcid.org/0000-0001-8483-3752
Civitello D https://orcid.org/0000-0001-8394-6288
Claidiere N https://orcid.org/0000-0002-4472-6597
Clark B
Clark E https://orcid.org/0000-0003-4289-6370
Clark N https://orcid.org/0000-0001-7131-3301
Clark R https://orcid.org/0000-0002-1100-1856
Clausen S
Clay Z https://orcid.org/0000-0002-3016-1732
Clemente C https://orcid.org/0000-0001-8174-3890
Clements C https://orcid.org/0000-0002-0637-1625
Clobert J
Cockburn A
Cole J
Colleary C https://orcid.org/0000-0003-2634-7355
Collet J
Collins C
Collins D
Colman E https://orcid.org/0000-0003-2551-8589
Colombelli-Negrel D
Colston T https://orcid.org/0000-0002-1127-1207
Coltman D
Comeault AA
Condamine F https://orcid.org/0000-0003-1673-9910
Cong P-Y https://orcid.org/0000-0001-9910-6478
Connallon T https://orcid.org/0000-0002-1962-4951
Conner J
Connor L
Conroy-Beam D https://orcid.org/0000-0003-3701-0870
Content A
Convey P https://orcid.org/0000-0001-8497-9903
Conway B
Cook C https://orcid.org/0000-0003-3310-2161
Cooke R
Coon K https://orcid.org/0000-0002-2701-5195
Cooney C https://orcid.org/0000-0002-4872-9146
Cooper C https://orcid.org/0000-0001-6225-2324
Corballis M
Cordero C
Corn K
Cornara D https://orcid.org/0000-0001-8258-2291
Cornelius J https://orcid.org/0000-0002-6334-7277
Cornell S
Cornwallis C
Corral Lopez A
Corsini M
Cortesi F https://orcid.org/0000-0002-7518-6159
Cory J https://orcid.org/0000-0002-4637-6439
Costello R
Cotto O https://orcid.org/0000-0003-0737-5879
Couch C
Coughlan J
Cowen L
Cox C https://orcid.org/0000-0002-9424-8482
Cox R
Coyte K
Cramer A
Cramer ER https://orcid.org/0000-0002-1443-9255
Cramp R
Cremer S
Crespi B https://orcid.org/0000-0002-0362-392X
Cressler C https://orcid.org/0000-0002-6281-2798
Crofts S https://orcid.org/0000-0003-1100-0009
Cronberg N
Cross P https://orcid.org/0000-0001-8045-5213
Crouch N https://orcid.org/0000-0002-3504-8245
Cruz S https://orcid.org/0000-0003-4775-8161
Crystal J
Cueva del Castillo R https://orcid.org/0000-0002-6385-0729
Culot L
Cummings M https://orcid.org/0000-0002-4873-5378
Currie C
Currie S https://orcid.org/0000-0001-5956-0481
Curtis N
Czenze Z
da Silva J https://orcid.org/0000-0001-5631-5421
Dahlin K
Dainat B
Dainese M https://orcid.org/0000-0001-7052-5572
Dakin R https://orcid.org/0000-0002-3140-3975
D'Alba L https://orcid.org/0000-0002-2478-3455
Dale J
D'Aloia C
Damer B
Daniel G
Daniel L
Daniel M
Daniel T https://orcid.org/0000-0002-5706-1096
Daniele C
Daniele P https://orcid.org/0000-0002-0122-479X
Dannemann M
Dargent F
Darolti I
Darroch S
Darwin S
Dasia S
Davenport RJ https://orcid.org/0000-0002-6828-9846
Daversa D
Dawson N https://orcid.org/0000-0001-5389-8692
Dawson W
de Bekker C http://orcid.org/0000-0002-7325-172X
De Bona S https://orcid.org/0000-0001-6193-7073
de Bruyn N https://orcid.org/0000-0002-9114-9569
De Deyn G https://orcid.org/0000-0003-4823-6912
De Dreu C https://orcid.org/0000-0003-3692-4611
de Guttry C
de Montaudouin X
De Petrillo F https://orcid.org/0000-0002-1289-2881
de Roos A
de Villemereuil P
De Wit P https://orcid.org/0000-0003-4709-3438
Dean M
Deangelis D
Dearborn D
Dearden P https://orcid.org/0000-0001-7790-9675
Debier C
DeCasien A https://orcid.org/0000-0002-6205-5408
Dechmann D https://orcid.org/0000-0003-0043-8267
Dee L https://orcid.org/0000-0003-0471-1371
Deffner D https://orcid.org/0000-0002-1649-3861
Dehning J https://orcid.org/0000-0002-1728-2505
Delerue F https://orcid.org/0000-0002-9809-5321
Delgado-Baquerizo M
Delhey K https://orcid.org/0000-0001-5190-5406
Denisov S
Derégnaucourt S
Derex M https://orcid.org/0000-0002-1512-6496
Dermauw W https://orcid.org/0000-0003-4612-8969
Desjonquères C https://orcid.org/0000-0002-6150-3264
Devigili A
Devos A
Devoto M
Dhaliwal N
Dhaval V
Dhevi K
Diamant E https://orcid.org/0000-0003-1727-5855
Díaz PG
Dickenson A
Diggle S https://orcid.org/0000-0003-3093-0412
DiLeo M https://orcid.org/0000-0003-0101-5274
Dimitrov D
Dingle C
Diogo R
Dixit T https://orcid.org/0000-0001-5604-7965
Dixson B https://orcid.org/0000-0003-0911-1244
Dolezal A https://orcid.org/0000-0001-6164-1344
Domenech de Cellès M https://orcid.org/0000-0002-9302-4858
Domínguez-Rodrigo M
Dominoni D https://orcid.org/0000-0003-2063-9955
Dominy N https://orcid.org/0000-0001-5916-418X
Donaldson J https://orcid.org/0000-0001-9267-3293
Donchin O
Donelan S https://orcid.org/0000-0002-4066-7884
Dores Cruz T https://orcid.org/0000-0002-4792-8469
Dorrough A
Doublet V
Dougherty L https://orcid.org/0000-0003-1406-0680
Douhard M https://orcid.org/0000-0001-9422-7270
Doutrelant C https://orcid.org/0000-0003-1893-3960
Dowdy N
Downing P https://orcid.org/0000-0002-5286-3153
Downs C
Drake J https://orcid.org/0000-0003-4646-1235
Dreyer D https://orcid.org/0000-0003-4344-8596
Drobniak S
Drumheller S
Drury J https://orcid.org/0000-0002-0262-8761
Drury J https://orcid.org/0000-0002-7748-5128
Duarte R https://orcid.org/0000-0001-7059-3129
Duchêne DA https://orcid.org/0000-0002-5479-1974
Duchenne F https://orcid.org/0000-0002-6917-8013
Duckworth R
Dudgeon S
Dudley S https://orcid.org/0000-0002-8660-3720
Dufour S
Dugas M
Dugdale H https://orcid.org/0000-0001-8769-0099
Dukas R https://orcid.org/0000-0003-4533-8542
Dunham A
Dunlop J https://orcid.org/0000-0002-0179-6640
Dunn D http://orcid.org/0000-0001-5909-1224
Dunn F
Dunn KJ http://orcid.org/0000-0003-0863-8757
Duperron S
Duport C
Dupoué A https://orcid.org/0000-0002-2501-464X
Durland E https://orcid.org/0000-0003-1322-6810
Dusek A https://orcid.org/0000-0001-8552-4089
Dussutour A https://orcid.org/0000-0002-1377-3550
Dutschmann M
DuVal E
Dwyer R
Dyble M https://orcid.org/0000-0001-6861-1631
Dyer A https://orcid.org/0000-0002-2632-9061
Dyer K https://orcid.org/0000-0001-7480-2055
Dylan M
Dziuba M
Eberhard W
Ecem AT
Edgar M
Edgecombe G https://orcid.org/0000-0002-9591-8011
Edgington M https://orcid.org/0000-0003-1922-9529
Eduardo CC
Edwards E
Edwin U
Edyta S https://orcid.org/0000-0003-1240-4814
Eggleton P https://orcid.org/0000-0002-1420-7518
Eguchi T
Ehrlén J
Eibye-Jacobsen D
Eikelboom J
Eisaguirre J https://orcid.org/0000-0002-0450-8472
Eisenberg D
Eisenberg E
Eisenhauer N
Ekroos J https://orcid.org/0000-0003-1164-5472
el Jundi B https://orcid.org/0000-0002-4539-6681
Elbanna A
Elias D https://orcid.org/0000-0002-5895-4275
Ellen J
Elliot S http://orcid.org/0000-0002-8973-9041
Elliott K https://orcid.org/0000-0001-5304-3993
Ellis R https://orcid.org/0000-0002-3117-0075
Elmhagen B
Emily B
Emily L
Emily S
Encinas-Viso F
Endler J https://orcid.org/0000-0002-7557-7627
Enríquez S https://orcid.org/0000-0001-5776-902X
Eric S
Erwann C
Evans B
Evans K
Ewan F
Ewelina R
Eyster H https://orcid.org/0000-0002-5571-3126
Ezard T
Fabbri G https://orcid.org/0000-0001-7690-8021
Fabre A-C
Fahey T
Falk J
Falkenberg L https://orcid.org/0000-0002-5868-2310
Falkingham P
Falkner A https://orcid.org/0000-0001-9756-7887
Faragó T https://orcid.org/0000-0001-5987-2629
Faria G https://orcid.org/0000-0002-1511-8680
Fariña R https://orcid.org/0000-0003-0898-0333
Farine D
Farrell MJ
Farris D https://orcid.org/0000-0002-6720-1961
Farwig N
Fattebert J https://orcid.org/0000-0001-5510-6804
Fawcett T https://orcid.org/0000-0001-6337-901X
Fayet A https://orcid.org/0000-0001-6373-0500
Federman S
Fedorenko E
Fedorka K
Feeney W https://orcid.org/0000-0002-7475-4724
Feilich K https://orcid.org/0000-0001-6057-897X
Feiner N https://orcid.org/0000-0003-4648-6950
Feldman M https://orcid.org/0000-0002-0664-3803
Feldmeyer B
Felmy A
Feng J
Fenton A
Ferguson L
Ferguson-Gow H
Fernandez F https://orcid.org/0000-0002-4288-5383
Fernandez R
Fernández-Marchán D
Ferrari J
Ferrari M
Ferreira G http://orcid.org/0000-0003-1554-8346
Ferrer-Obiol J
Ferrier D
Ferriera S
Ferrigno S
Ferris D http://orcid.org/0000-0001-6373-6021
Ferris K
Ferrón H https://orcid.org/0000-0003-2254-8424
Festa-Bianchet M
Feulner P
Fey S https://orcid.org/0000-0002-7471-3308
Fias W
Fichtel C https://orcid.org/0000-0002-8346-2168
Fidino M
Fietzke J
Figueroa L https://orcid.org/0000-0003-0655-8278
Fine M https://orcid.org/0000-0001-9665-1462
Finet C
Fiorito G
Fischer A https://orcid.org/0000-0001-5922-8411
Fischer E https://orcid.org/0000-0002-2916-0900
Fischhoff I https://orcid.org/0000-0001-6956-8284
Fisher D
Fisher D
Fisher J
Fisher R https://orcid.org/0000-0002-2956-3240
Fitton L
Fitzgibbon L https://orcid.org/0000-0002-8563-391X
Fjeldså J
Flanagan L
Fleming R
Flexas Sans J
Fogelström E https://orcid.org/0000-0002-5755-849X
Foitzik S https://orcid.org/0000-0001-8161-6306
Folk R https://orcid.org/0000-0002-5333-9273
Fones H https://orcid.org/0000-0002-9914-4885
Foo S https://orcid.org/0000-0002-7083-2377
Ford B
Forde T
Forrest J
Forrister D
Forthman M
Fortin D https://orcid.org/0000-0003-1267-1891
Fossette S
Foster B https://orcid.org/0000-0002-1467-0078
Fournier G https://orcid.org/0000-0003-1605-5455
Fox J https://orcid.org/0000-0001-6374-657X
Fox-Dobbs K
Fragaszy D
Francis J
Francisca P
Frank S https://orcid.org/0000-0001-7348-7794
Franks N https://orcid.org/0000-0001-8139-9604
Frantsevich L
Frantz L http://orcid.org/0000-0001-8030-3885
Fraser D https://orcid.org/0000-0002-0228-2617
Freckleton R https://orcid.org/0000-0002-8338-864X
Frederickson M https://orcid.org/0000-0002-9058-7137
Freeman B
Frentiu FD https://orcid.org/0000-0001-8628-4216
Frere C https://orcid.org/0000-0002-9671-2138
Fretwell P
Friberg U https://orcid.org/0000-0001-6112-9586
Frid A
Friedman N https://orcid.org/0000-0002-0533-6801
Friman V-P
Fritz-Laylin L
Fromhage L https://orcid.org/0000-0001-5560-6673
Fronhofer E
Fruciano C
Frye B
Fryxell J https://orcid.org/0000-0002-5278-8747
Fu F
Fu Y
Fuenzalida T
Fumiaki N
Fumiaki YN
Furukawa S
Fusco G https://orcid.org/0000-0002-4690-6049
Fuxjager M https://orcid.org/0000-0003-0591-6854
Fuzessy L https://orcid.org/0000-0001-9599-9782
Gabriela Š
Gagliardo A https://orcid.org/0000-0003-0214-3817
Gahr M https://orcid.org/0000-0002-6602-2291
Gaillard J-M
Galati E
Galaverni M
Galetti M
Galiana N https://orcid.org/0000-0001-7720-0615
Gallagher C https://orcid.org/0000-0001-7094-1752
Gallup A
Galván-Magaña F
Gamboa M
Gamperl K
Gan YH
Gaos A https://orcid.org/0000-0001-6100-7319
Garcia J
Gardner A https://orcid.org/0000-0002-1304-3734
Garnier A
Garvin M
Gauzere J
Geange S
Gearty W https://orcid.org/0000-0003-0076-3262
Geber M
Gee B https://orcid.org/0000-0003-4517-3290
Gegenfurtner K
Geisler J
Gems D https://orcid.org/0000-0002-6653-4676
Genner M
Georgeson M
Germain R https://orcid.org/0000-0002-1270-6639
German D
Gershman S https://orcid.org/0000-0003-4451-820X
Ghoshal R
Gibson B
Gibson B
Gibson E
Gibson M
Gienapp P
Gifford M https://orcid.org/0000-0003-1263-0010
Gil M
Gilbert A https://orcid.org/0000-0002-7632-8550
Gilbert J https://orcid.org/0000-0001-7014-2803
Gilbert K
Gilby B https://orcid.org/0000-0001-8642-9411
Gill S https://orcid.org/0000-0002-4628-8922
Gillis A https://orcid.org/0000-0003-2062-3777
Gilman R https://orcid.org/0000-0003-2000-7966
Gilor O
Gimenez L https://orcid.org/0000-0002-1472-2915
Girard F
Girard-Buttoz C https://orcid.org/0000-0003-1742-4400
Girardet J
Glazier D https://orcid.org/0000-0001-7164-1823
Glen A
Goberna M
Goddard M
Godoy P https://orcid.org/0000-0003-4519-5094
Gols R https://orcid.org/0000-0002-6839-8225
Gómez-Bahamón V
Gómez-Brandón M
Gómez-Laich A
Goncalves A https://orcid.org/0000-0001-7062-1900
González-Forero M https://orcid.org/0000-0003-1015-3089
Gonzalez-Gomez P
Gonzalez-Rodriguez D https://orcid.org/0000-0001-6152-2222
Gonzalez-Voyer A
Goodman S
Goodwin C
Gopalaswamy A https://orcid.org/0000-0003-3841-3663
Gopko M
Gordon D https://orcid.org/0000-0002-1090-9539
Gordon Danner S https://orcid.org/0000-0001-7749-9127
Gorjanc G https://orcid.org/0000-0001-8008-2787
Gosler A
Goss G
Gotoh H
Gottdenker N
Gotthard K
Goupil L
Govaert L https://orcid.org/0000-0001-8326-3591
Gow E https://orcid.org/0000-0001-8890-4503
Grace S
Graham A
Graham K https://orcid.org/0000-0001-9445-0202
Graham P
Granath GK https://orcid.org/0000-0002-3632-9102
Granatosky M https://orcid.org/0000-0002-6465-5386
Graniero LE
Grass I
Gratwicke B
Gray P
Graystock P https://orcid.org/0000-0002-0248-2571
Green A
Green C
Green D
Green R https://orcid.org/0000-0001-8690-8914
Green S https://orcid.org/0000-0002-2208-5450
Greenberg D https://orcid.org/0000-0001-7489-1393
Greenlee K
Grether G https://orcid.org/0000-0003-3441-0537
Grieshop K https://orcid.org/0000-0001-8925-5066
Griesser M
Griffen B https://orcid.org/0000-0002-8126-6323
Griffith S
Griffiths J https://orcid.org/0000-0003-0319-515X
Griggo C
Grigoratou M https://orcid.org/0000-0003-0484-1408
Groenen M
Grogan K
Grove M
Grueber C https://orcid.org/0000-0002-8179-1822
Grunberg R https://orcid.org/0000-0001-9926-4978
Grunst M https://orcid.org/0000-0002-3425-4020
Grüter C
Grutter A
Guerra PA
Guglielmo C
Guidetti R
Guillaume F
Guillette L https://orcid.org/0000-0002-8777-6543
Guindre-Parker S https://orcid.org/0000-0002-6205-3752
Gurarie E https://orcid.org/0000-0002-8666-9674
Gustison M
Gutiérrez J https://orcid.org/0000-0001-8459-3162
Gutierrez-Ibanez C https://orcid.org/0000-0002-0468-8223
Guzman LM
Gwynne D
Haag C
Haase C
Hadjivasiliou Z https://orcid.org/0000-0003-1174-1421
Haegeman B
Hager F
Hairston N
Hall D
Hall J https://orcid.org/0000-0002-4896-4592
Hall R
Hamelin F
Hamilton D https://orcid.org/0000-0001-5883-0136
Hammer T https://orcid.org/0000-0002-7308-8440
Hammerschmidt K
Han B https://orcid.org/0000-0002-9948-3078
Han C https://orcid.org/0000-0001-9261-4031
Hancock P
Handegard NO
Handley C
Hanley D https://orcid.org/0000-0003-0523-4335
Hanlon R https://orcid.org/0000-0003-0004-5674
Hanmer H https://orcid.org/0000-0001-5933-7554
Hannah D
Hannelore M
Harborne A
Hardy O
Hare M
Haridy Y https://orcid.org/0000-0002-6908-5749
Harper D
Harper J
Harris J
Harris R
Harrison J https://orcid.org/0000-0001-5223-216X
Harrison X https://orcid.org/0000-0002-2004-3601
Hart D
Hart T https://orcid.org/0000-0002-4527-5046
Hartstone-Rose A
Harvell D
Harvey B
Harvey M
HARVEY T https://orcid.org/0000-0002-2717-7004
Hasenjager M https://orcid.org/0000-0003-2193-4120
Hashimoto Y
Hatala K https://orcid.org/0000-0001-9131-5304
Havird J https://orcid.org/0000-0002-8692-6503
Hawkins MB
Hawlena D
Hawlena H https://orcid.org/0000-0002-0634-2920
Hawley D
Hay M
Hayes L https://orcid.org/0000-0003-0713-416X
Haynes P
Hays CG
Hayward A https://orcid.org/0000-0001-6953-7509
Hayward MW https://orcid.org/0000-0002-5574-1653
Hazkani-Covo E
Heatwole H
Hébert K
Hebets E https://orcid.org/0000-0002-9382-2040
Heckman R https://orcid.org/0000-0002-2281-3091
Hedrick T https://orcid.org/0000-0002-6573-9602
Heesy C
Heffner J
Heidinger B https://orcid.org/0000-0003-0064-209X
Heil C
Heilpern S
Heinrichs JA
Heisenberg C-P
Helantera H https://orcid.org/0000-0002-6468-5956
Helder J
Heleno R
Helm B
Hempson G
Henley M
Hennig F
Henningsson P https://orcid.org/0000-0003-2640-1067
Henshaw J https://orcid.org/0000-0001-7306-170X
Herbert-Read J https://orcid.org/0000-0003-0243-4518
Hernandez P
Hernández-Gómez O
Hernik M
Herrando-Pérez S https://orcid.org/0000-0001-6052-6854
Herre A
Herrel A
Herrera-Alsina L https://orcid.org/0000-0003-0474-3592
Hertel F
Hess U https://orcid.org/0000-0002-6793-7282
Hesse E https://orcid.org/0000-0002-1900-7136
Heubel K
Heyland A https://orcid.org/0000-0002-7592-4473
Heymann E https://orcid.org/0000-0002-4259-8018
Heywood J
Hickler T
Hietz P
Higgs D https://orcid.org/0000-0002-0771-4642
Hillebrand H https://orcid.org/0000-0001-7449-1613
Hillen T
Hirsch B
Hladish T
Ho S
Hobson K
Hodge J https://orcid.org/0000-0003-1603-8559
Hodson-Tole E https://orcid.org/0000-0003-1200-1724
Hoelzel R https://orcid.org/0000-0002-7265-4180
Hoey J
Hoffman P
Hoi A https://orcid.org/0000-0002-7477-5269
Holekamp K
Holen S
Holl K
Holliday T
Hollingsworth P
Holman L https://orcid.org/0000-0002-7268-2173
Holmes JD https://orcid.org/0000-0001-8804-2149
Holmes M
Holovachov O https://orcid.org/0000-0002-4285-0754
Holt N
Holt R https://orcid.org/0000-0002-6685-547X
Holzer AS https://orcid.org/0000-0002-4916-3172
Holzman R
Hoogenboom M
Hopkins B https://orcid.org/0000-0002-9760-6185
Hopkins MJ https://orcid.org/0000-0002-3580-2172
Hopkins R https://orcid.org/0000-0002-6283-4145
Hopper G https://orcid.org/0000-0001-6990-1681
Houslay T https://orcid.org/0000-0001-5592-9034
Houssaye A
How M https://orcid.org/0000-0001-5135-8828
Howell N
Hrcek J https://orcid.org/0000-0003-0711-6447
Hsieh C-H
Huang H-T
Huang S https://orcid.org/0000-0002-5055-1308
Huang Z
Huckstadt L https://orcid.org/0000-0002-2453-7350
Hudson P
Hulme P
Hund A
Hung C-M
Hunter M
Husby A https://orcid.org/0000-0003-1911-8351
Hut R https://orcid.org/0000-0003-4552-0985
Hwang P-P https://orcid.org/0000-0002-5534-2812
Ichie T
Ihara Y https://orcid.org/0000-0003-3072-2410
Ilany A https://orcid.org/0000-0001-8089-569X
Ilya M
Imai H
Imhof L
Imperial J
Ip JC-H https://orcid.org/0000-0002-4477-7351
Iranzo J
Iritani R https://orcid.org/0000-0002-2396-1109
Irwin D https://orcid.org/0000-0002-1050-5841
Isanta-Navarro J https://orcid.org/0000-0002-6168-4499
Ishii H
Itescu Y
Jack K
Jack S
Jackson M
Jacob E
Jacobs A
Jagannath A
Jakobsen L https://orcid.org/0000-0002-8496-5576
James HG
Jamie C
Jamie G https://orcid.org/0000-0002-2766-7687
Janet C
Janiak M
Jansa S
Jara-Ettinger J https://orcid.org/0000-0002-6167-1647
Jarvis E
Jasmine H
Jason T
Jassey V
Jean-Phillippe C
Jeffries D https://orcid.org/0000-0003-1701-3978
Jenni L
Jenouvrier S
Jensen O
Jensen S
Jessica B
Jessica L-D https://orcid.org/0000-0002-1088-8766
Jessica R
Jiang G https://orcid.org/0000-0003-1901-4480
Jimeno B https://orcid.org/0000-0003-3040-0163
Jingyuan C
Jockusch E
Johnson L
Johnson MA https://orcid.org/0000-0002-9278-5639
Johnson R https://orcid.org/0000-0002-2431-0180
Johnson S https://orcid.org/0000-0002-5114-5862
Jolles J https://orcid.org/0000-0001-9905-2633
Jonas V
Jones H
Jones P https://orcid.org/0000-0001-7147-7699
Jongerius C
Jordan A https://orcid.org/0000-0001-6131-9734
Jordan F
José B
Joseph G https://orcid.org/0000-0002-8731-9828
Joseph L https://orcid.org/0000-0001-7564-1978
Julia S
Jung J-L https://orcid.org/0000-0002-8795-8056
Jusufi A
Kamath P
Kamikouchi A https://orcid.org/0000-0003-1552-6892
Kamimura A https://orcid.org/0000-0001-9934-8688
Kammenga J
Kandlikar G https://orcid.org/0000-0003-3043-6780
Kane A
Kang C-K
Kang L
Kaplan H https://orcid.org/0000-0002-8583-5714
Kapli P
Kara M
Karasov W
Kardos M https://orcid.org/0000-0002-7587-3004
Karthik Y
Kasha S
Kasumyan A
Katabuchi M
Kathleen G
Kathleen H
Kathleen N
Kato A
Katrina C
Katsuki M
Kaul R
Kaur K
Kavanagh E https://orcid.org/0000-0001-7202-005X
Kavanagh K https://orcid.org/0000-0002-1496-990X
Kawecki TJ https://orcid.org/0000-0002-9244-1991
Ke P-J
Keaney T
Kearney S
Keeling C
Keertana T
Keesey I
Kehlmaier C https://orcid.org/0000-0001-9622-0566
Kelber A https://orcid.org/0000-0003-3937-2808
Keller L
Keller P
Kellermann V https://orcid.org/0000-0002-9859-9642
Kelly A http://orcid.org/0000-0002-6013-3943
Kelly J https://orcid.org/0000-0001-9480-1252
Kelly M https://orcid.org/0000-0001-6998-5053
Kelsey A-B
Kendra B
Kenkel C
Kennedy D https://orcid.org/0000-0003-0820-115X
Kennedy G
Kennedy J
Kennedy P https://orcid.org/0000-0002-2524-6192
Kenny N https://orcid.org/0000-0003-4816-4103
Kerr J https://orcid.org/0000-0002-3972-7560
Kershenbaum A
Keupp S
Khaliq I
Khan I https://orcid.org/0000-0002-0110-2274
Khannoon E
Khila A https://orcid.org/0000-0003-0908-483X
Khudyakov J https://orcid.org/0000-0001-7038-102X
Kikuchi D https://orcid.org/0000-0002-7379-2788
Kilbourne B
Kim H
Kim H
Kim S-Y https://orcid.org/0000-0002-5170-8477
Kim T
Kim W
Kimball R
King K
Kingma S
Kingman G
Kinlock N https://orcid.org/0000-0002-2917-5133
Kirk D
Kirschel A https://orcid.org/0000-0003-4379-7956
Kirstin C
Kissane R https://orcid.org/0000-0001-9385-2584
Kitano J https://orcid.org/0000-0001-8659-5698
Kivell T
Kjellberg F
Kjernsmo K https://orcid.org/0000-0002-8570-0406
Klein C https://orcid.org/0000-0001-6485-5399
Kleindorfer S https://orcid.org/0000-0001-5130-3122
Klinck J
Klug C https://orcid.org/0000-0002-4099-7453
Klug H https://orcid.org/0000-0003-3169-2452
Klümper U https://orcid.org/0000-0002-4169-6548
Knief U https://orcid.org/0000-0001-6959-3033
Knight E
Knörnschild M https://orcid.org/0000-0003-0448-9600
Knudsen E
Kobayashi D
Kocsis Á https://orcid.org/0000-0002-9028-665X
Kodandaramaiah U https://orcid.org/0000-0002-1564-1738
Koella J
Koenig D
Kohei T
Kohl K
Kokko H https://orcid.org/0000-0002-5772-4881
Koleček J https://orcid.org/0000-0003-1069-6593
Kondoh M
Korb J
Kortessis N
Koski M https://orcid.org/0000-0002-0274-3145
Kössl M
Koteja P https://orcid.org/0000-0003-0077-4957
Kotrschal A https://orcid.org/0000-0003-3473-1402
Kozak G
Kozakiewicz CP
Kraaijeveld K
Krabbenhoft T
Kramer K https://orcid.org/0000-0002-9157-7758
Krams I https://orcid.org/0000-0001-7150-4108
Kratochvil L https://orcid.org/0000-0002-3515-729X
Kraus F
Kret M https://orcid.org/0000-0002-3197-5084
Krishnan A https://orcid.org/0000-0002-8317-4991
Kristiana B
Kröger B https://orcid.org/0000-0002-2427-2364
Kronauer D
Krug J https://orcid.org/0000-0002-2143-6490
Krupp D Brian
Krzywinski J
Ksepka D
Ku C https://orcid.org/0000-0001-6414-4423
Kuhlwilm M https://orcid.org/0000-0002-0115-1797
Kuhn J
Kulahci I https://orcid.org/0000-0003-0104-0365
Kung K
Kupriyanova E
Kurata J
Kutsukake M
Kutsukake N https://orcid.org/0000-0001-8637-6233
Labandeira C
Laberge F
Labonte D https://orcid.org/0000-0002-1952-8732
Lachlan R
LaCroix T
Ladich F
Lafferty K https://orcid.org/0000-0001-7583-4593
Lagisz M
Lagrue C
Laibl L
Lailvaux S
LaJeunesse T
Lamb E
Lambert M https://orcid.org/0000-0003-2318-6445
Lambrecht S
Lameira A https://orcid.org/0000-0003-3071-4824
Laming S
Lammers M https://orcid.org/0000-0003-3184-1825
Landi P https://orcid.org/0000-0002-6373-2992
Landi S
Lanfear R
Larouche O
Larsen E
Larson G https://orcid.org/0000-0002-4092-0392
Lasky J https://orcid.org/0000-0001-7688-5296
Latombe G
Laura J
Lauren G
Lauterbur ME
Lavery J https://orcid.org/0000-0003-1179-1445
Law P https://orcid.org/0000-0003-3382-0286
Lawver D
Layton K
Leão P
LeBlanc A https://orcid.org/0000-0002-2497-1296
Lee A
Lee A
Lee C-R https://orcid.org/0000-0002-1913-9964
Lee P
Leech T
Lees A
Legett H https://orcid.org/0000-0001-9005-8641
Lehmann G
Lehmann J
Lehmann L
Lehtonen J https://orcid.org/0000-0001-5260-1041
Lehtonen T https://orcid.org/0000-0002-1372-9509
Leitner S https://orcid.org/0000-0002-9482-0362
Leiva F
Lemasson B https://orcid.org/0000-0001-8022-110X
Lemmel-Schaedelin F
Lenormand T https://orcid.org/0000-0001-8930-5393
Lepczyk C https://orcid.org/0000-0002-5316-3159
Lev-Ari S https://orcid.org/0000-0002-3784-5184
Levin I https://orcid.org/0000-0001-8932-5773
Levy M
Lewis JJ
Lewis L https://orcid.org/0000-0003-1693-5065
Lewis R
Li C https://orcid.org/0000-0001-7516-3646
Li D https://orcid.org/0000-0001-8269-7734
Li J
Li R
Li Y
Li Y https://orcid.org/0000-0002-2281-4529
Li Y https://orcid.org/0000-0002-5258-877X
Liana W
Liao J https://orcid.org/0000-0002-9520-3235
Libbrecht R https://orcid.org/0000-0003-4397-000X
Libby N
Lichtwark G
Liesbeth VH
Lily B
Lima M https://orcid.org/0000-0002-3700-2945
Lin D
Lin S
Lindmark M
Lindo Z
Lindqvist C https://orcid.org/0000-0002-4190-727X
Lindstedt C https://orcid.org/0000-0001-8176-3613
Ling N
Linksvayer T
Linnen C
Lisovski S
List J-M https://orcid.org/0000-0003-2133-8919
Little C http://orcid.org/0000-0003-2803-7465
Liu B
Liu X https://orcid.org/0000-0002-9292-4432
Liu Y
Llaurens V
Lloyd-Smith J
Lo N
Lobmaier J https://orcid.org/0000-0001-8025-1616
Lodjak J https://orcid.org/0000-0001-8089-948X
Loe LE
Lofgren E https://orcid.org/0000-0003-2052-7240
Loiseau O https://orcid.org/0000-0002-9852-857X
Longo A https://orcid.org/0000-0002-5112-1246
Longrich N https://orcid.org/0000-0002-9586-8907
Lonsdorf E https://orcid.org/0000-0001-8057-401X
Lopez-Uribe MM https://orcid.org/0000-0002-8185-2904
Lord J http://orcid.org/0000-0001-6616-1526
Lorenz A
Lorenz T
Lorenzo Merino C
Lotterhos K https://orcid.org/0000-0001-7529-2771
Louder M https://orcid.org/0000-0003-4421-541X
Louys J https://orcid.org/0000-0001-7539-0689
Lu M https://orcid.org/0000-0002-4949-8837
Luberti F
Lucy T
Ludington W
Luiz O https://orcid.org/0000-0002-6995-6524
Lukas D https://orcid.org/0000-0002-7141-3545
Lund-Hansen K
Lunn T http://orcid.org/0000-0003-4439-2045
Luo J https://orcid.org/0000-0002-2780-475X
Lupyan G https://orcid.org/0000-0001-8441-7433
Lyberger K https://orcid.org/0000-0003-4903-380X
Lymbery R https://orcid.org/0000-0001-8041-6169
Lynch H https://orcid.org/0000-0002-9026-1612
Lynda B
Ma X https://orcid.org/0000-0002-1172-6611
Mac Nally R
Macartney E https://orcid.org/0000-0003-3866-143X
MacColl A https://orcid.org/0000-0003-2102-6130
Mackenzie U-C
Mackessy S https://orcid.org/0000-0003-4515-2545
Maclean I https://orcid.org/0000-0001-8030-9136
MacMillan H https://orcid.org/0000-0001-7598-3273
MacPherson A
Madeline M
Madhusudhana S https://orcid.org/0000-0002-4142-3881
Madsen T https://orcid.org/0000-0002-0998-8372
Maggi E http://orcid.org/0000-0001-6934-9380
Magrach A https://orcid.org/0000-0003-2155-7556
Mahon M
Maja D
Makin A https://orcid.org/0000-0002-4490-7400
Makoto K
Małgorzata L
Małgorzata L https://orcid.org/0000-0003-3550-4105
Malhi R
Malicki M
Mallet J
Mamassian P
Manas S
Manel S
Manlove K https://orcid.org/0000-0002-7200-5236
Manuel B
Marco A
Marcus C
Margos G
Marianne E
Maried P
Marina P
Marini L
Marino JA
Marinovic W https://orcid.org/0000-0002-2472-7955
Mario M
Marion G https://orcid.org/0000-0002-0454-9338
Marion S
Markert S
Markham C
Marler C
Marlétaz F
Marshall D http://orcid.org/0000-0003-3771-5950
Marshall H
Marshall H https://orcid.org/0000-0003-2120-243X
Martin B
Martin D https://orcid.org/0000-0001-6350-7384
Martin J https://orcid.org/0000-0001-9159-645X
Martin K
Martin M https://orcid.org/0000-0002-3556-6632
Martin R
Martin R
Martin S https://orcid.org/0000-0002-0747-7456
Martínez-Abraín A https://orcid.org/0000-0001-8009-4331
Martinez-Cerón MC
Martin-Loeches M
Maruyama P
Marx F
Mason A https://orcid.org/0000-0001-7719-8500
Mason G https://orcid.org/0000-0001-8045-4579
Matassa C https://orcid.org/0000-0003-2632-6191
Mathur S
Matos F https://orcid.org/0000-0002-7699-9522
Matsumura Y https://orcid.org/0000-0002-3438-2161
Matsuo T https://orcid.org/0000-0002-4185-6740
Matthew M
Matthews B
Matthews P https://orcid.org/0000-0003-0682-8522
Mattison S https://orcid.org/0000-0002-9537-5459
Matute D
Matz M
Maureaud A https://orcid.org/0000-0003-4778-9443
Mayerl C https://orcid.org/0000-0003-0402-8388
Mazza V https://orcid.org/0000-0002-1634-3417
McCann K
McClelland G
McCoy D https://orcid.org/0000-0001-8383-8084
McCulloch K https://orcid.org/0000-0002-6203-7381
McCullough ME
McDonald B https://orcid.org/0000-0001-5028-066X
McDonald M http://orcid.org/0000-0002-5735-960X
McFarlane E
McGlothlin J https://orcid.org/0000-0003-3645-6264
McGregor P
McGuire J
McHenry M https://orcid.org/0000-0001-5834-674X
McKenna K
Mckenzie V http://orcid.org/0000-0002-9940-9556
McKinney G
McLachlan J https://orcid.org/0000-0001-6275-7971
McLennan D
McLeod A
McLeod D https://orcid.org/0000-0003-2551-7877
McMahon W https://orcid.org/0000-0003-2174-1695
McNamara J https://orcid.org/0000-0002-4235-3045
Medina I https://orcid.org/0000-0002-1021-5035
Meek M
Meeus I
Megan K
Meier J
Meijer J https://orcid.org/0000-0001-6619-5312
Meineke EK
Meirmans P
Mello M https://orcid.org/0000-0002-9098-9427
Memmott J
Mendez-Rojas D
Mendl M https://orcid.org/0000-0002-5302-1871
Menge B
Menke S
Menzel R
Mergeay J https://orcid.org/0000-0002-6504-0551
Merilä J https://orcid.org/0000-0001-9614-0072
Merilaita S https://orcid.org/0000-0001-6437-9863
Mérot C http://orcid.org/0000-0003-2607-7818
Merrill R
Merrill T
Merritt J
Meseguer A
Mesoudi A https://orcid.org/0000-0002-7740-1625
Messier C
Metcalfe N https://orcid.org/0000-0002-1970-9349
Mettke-Hofmann C
Metz D
Michael C
Michael Shuker D
Michaud J https://orcid.org/0000-0001-6194-1355
Michelangeli M
Michelsen A
Michez D https://orcid.org/0000-0001-8880-1838
Michod R https://orcid.org/0000-0002-7782-0379
Mideo N
Mielke A https://orcid.org/0000-0002-8847-6665
Miettinen T https://orcid.org/0000-0002-5975-200X
Mikhlina A https://orcid.org/0000-0002-9231-0545
Mila B
Milla R
Millar J
Miller E
Miller M
Miller P
Mills S https://orcid.org/0000-0001-8948-3384
Milot E
Milward S
Mina O
Miranda W
Mirth C https://orcid.org/0000-0002-9765-4021
Mitchell D https://orcid.org/0000-0002-6008-2672
Mitchell G
Mitchell J
Mizukoshi A
Møbjerg N
Mocenni C
Mock D
Moeller D
Moffett E
Mogan R
Mohr S https://orcid.org/0000-0002-9089-6327
Moldovan O
Moleón M
Moles A https://orcid.org/0000-0003-2041-7762
Molleman L
Mona LL
Monaco C
Monaghan P https://orcid.org/0000-0003-2430-0326
Mondloch C https://orcid.org/0000-0002-6238-1238
Mongiardino Koch N https://orcid.org/0000-0001-6317-5869
Monika Y
Montealegre-Z F https://orcid.org/0000-0001-5186-2186
Monteiro T https://orcid.org/0000-0002-2836-8961
Montiglio P-O
Mooers A
Mooney TA https://orcid.org/0000-0002-5098-3354
Moore A https://orcid.org/0000-0002-1498-3322
Moosmann M
Morand S https://orcid.org/0000-0003-3986-7659
Morandin C
Morant J
Morante-Filho J
Morash A
Mordecai E https://orcid.org/0000-0002-4402-5547
Moreno Azócar D https://orcid.org/0000-0002-8616-0544
Moret Y https://orcid.org/0000-0002-2435-8416
Morey C https://orcid.org/0000-0001-9513-0278
Morganti T https://orcid.org/0000-0001-6928-9496
Mori A https://orcid.org/0000-0002-8422-1198
Mori Y https://orcid.org/0000-0002-2216-0190
Morimoto J https://orcid.org/0000-0003-3561-1920
Morley V https://orcid.org/0000-0001-6805-7562
Morris R http://orcid.org/0000-0001-8661-1520
Morrison E
Mos B https://orcid.org/0000-0003-3687-516X
Motta S https://orcid.org/0000-0002-2038-529X
Mourier J https://orcid.org/0000-0001-9019-1717
Mouton J https://orcid.org/0000-0002-8475-9078
Mueller C
Mueller JL
Müller N https://orcid.org/0000-0001-5213-042X
Mullon C https://orcid.org/0000-0002-9875-4227
Mumford J
Munday P
Murali G https://orcid.org/0000-0002-5307-325X
Murphy D
Mutanen M
Nadine S
Naeem S https://orcid.org/0000-0002-6569-2648
Nagelkerken I https://orcid.org/0000-0003-4499-3940
Nagler J
Nakayama S
Narendra A https://orcid.org/0000-0002-1286-5373
Nash M
Natalie I
Natasha S
Nathan W
Nathaniel Z
Nauen R
Nava E
Nawroth C https://orcid.org/0000-0003-4582-4057
Naya D https://orcid.org/0000-0002-8311-9263
Nelson D
Nelson M
Nelson X https://orcid.org/0000-0003-4301-2928
Nespolo R https://orcid.org/0000-0001-9337-0606
Ness R https://orcid.org/0000-0001-7313-8236
Nettle D https://orcid.org/0000-0001-9089-2599
Nevo O https://orcid.org/0000-0003-3549-4509
Newbery D https://orcid.org/0000-0002-0307-3489
Newton-Fisher N
Nick M
Nicole B
Niels P
Niels P
Nielsen B
Nielsen M
Niemi G
Nietlisbach P https://orcid.org/0000-0002-6224-2246
Nilsson GE
Nimmo D https://orcid.org/0000-0002-9814-1009
Niu K https://orcid.org/0000-0003-4845-2930
Noble L https://orcid.org/0000-0002-5161-4059
Noë R
Noge K
Nolet B
Noor S
Norberg A
Nord A https://orcid.org/0000-0001-6170-689X
Noren D https://orcid.org/0000-0002-9544-4530
Norris D https://orcid.org/0000-0003-4874-1425
Novelletto A https://orcid.org/0000-0002-1146-7680
Nowotny M https://orcid.org/0000-0002-2854-6908
Nunn C https://orcid.org/0000-0001-9330-2873
Nussaïbah RS
Nyakatura J https://orcid.org/0000-0001-8088-8684
OConnor P https://orcid.org/0000-0002-6762-3806
Octavia B
Ode P
O'Dea A https://orcid.org/0000-0001-5495-4764
O'Dea R https://orcid.org/0000-0001-8177-5075
Odemer R https://orcid.org/0000-0003-2230-4294
Ogutcen E
O'Hara R https://orcid.org/0000-0001-9737-3724
Ohmer M https://orcid.org/0000-0002-5937-6585
Okanishi M
Oke K
Oldroyd B https://orcid.org/0000-0001-6831-3677
Olito C https://orcid.org/0000-0001-6883-0367
Oliver C
Oliver K
Oliver T
Olson S https://orcid.org/0000-0002-8484-9006
Olsson M https://orcid.org/0000-0002-4130-1323
O'Neill M https://orcid.org/0000-0001-9614-7813
Oono R https://orcid.org/0000-0003-4859-0030
Ophir A https://orcid.org/0000-0002-4877-9696
Oppel S
Ord T https://orcid.org/0000-0002-2608-2150
O'Regan S
Orizaola G
Orkin J
Orsini L
Ortega G https://orcid.org/0000-0001-5305-1467
Ortega J
Ortega-Hernández J https://orcid.org/0000-0002-6801-7373
Orteiza P
Orton R https://orcid.org/0000-0002-3389-4325
Osenberg C https://orcid.org/0000-0003-1918-7904
Osmond M
Ostevik K https://orcid.org/0000-0002-2197-9284
Otti O https://orcid.org/0000-0002-2361-9661
Otto S https://orcid.org/0000-0003-3042-0818
Oufiero C
Ouyang J
Owen CD
Owen-Smith N
Padfield D
Pagán I https://orcid.org/0000-0001-8876-1194
Pagano A
Page A https://orcid.org/0000-0002-0973-1569
Paitz R https://orcid.org/0000-0003-4609-4359
Pakanen V-M
Palacio F
Palagi E https://orcid.org/0000-0002-2038-4596
Palaoro A https://orcid.org/0000-0002-8629-0728
Palmer AR
Panos B
Paquet M https://orcid.org/0000-0003-1182-2299
Park A https://orcid.org/0000-0003-4080-7274
Park T https://orcid.org/0000-0002-9492-8859
Park T-Y https://orcid.org/0000-0002-8985-930X
Parker B
Parks S https://orcid.org/0000-0001-6663-627X
Parravicini V https://orcid.org/0000-0002-3408-1625
Parreira B https://orcid.org/0000-0002-5673-4199
Parry L
Parsch J
Paschalis N
Passamonti M
Patel A https://orcid.org/0000-0003-2229-9341
Pates S https://orcid.org/0000-0001-8063-9469
Patten M
Patton A https://orcid.org/0000-0003-1286-9005
Paul C
Paulk A
Pauly D
Peak C https://orcid.org/0000-0001-6150-5318
Pearse D
Pearse I https://orcid.org/0000-0001-7098-0495
Pedersen E
Peel A
Peer B https://orcid.org/0000-0002-4423-0508
Peguero G https://orcid.org/0000-0002-6464-1486
Peixoto PE https://orcid.org/0000-0003-4127-625X
Pekar S https://orcid.org/0000-0002-0197-5040
Pelegri J
Pelletier F
Penacchio O https://orcid.org/0000-0002-1544-2405
Pepperberg I https://orcid.org/0000-0001-9486-7323
Perc M https://orcid.org/0000-0002-3087-541X
Perez T http://orcid.org/0000-0002-3707-7285
Perez-Lamarque B https://orcid.org/0000-0001-7112-7197
Pernet B
Perrard A http://orcid.org/0000-0002-4279-9987
Perri A
Perrier V
Perrot T
Perry C https://orcid.org/0000-0001-9398-2418
Petchey O
Peter H
Peters R https://orcid.org/0000-0002-5825-3591
Phifer-Rixey M
Phillimore A https://orcid.org/0000-0002-6553-1553
Phillips M
Phillips RD
Phoebe E
Piano E
Piazza M
Piccolo JJ
Pick JL https://orcid.org/0000-0002-6295-3742
Pickett J https://orcid.org/0000-0002-8386-3770
Pierce S
Pierre-Olivier M
Pigeault R
Pilastro A https://orcid.org/0000-0001-9803-6308
Pimiento C https://orcid.org/0000-0002-5320-7246
Pineda-Munoz S
Pinheiro H https://orcid.org/0000-0002-3143-1474
Pinotti F
Pires MM https://orcid.org/0000-0003-2500-4748
Pirk C https://orcid.org/0000-0001-6821-7044
Pirrie A https://orcid.org/0000-0001-9489-4401
Pitts-Singer T
Pitzer V
Pizo M
Plaistow S https://orcid.org/0000-0002-9003-6271
Podos J
Poelstra J
Pohjoismäki J https://orcid.org/0000-0002-1185-3610
Pokorny L
Polidori G
Polly P
Pomiechowska B https://orcid.org/0000-0002-3819-7641
Pondorfer A https://orcid.org/0000-0001-8435-7493
Ponganis P
Pongracz P https://orcid.org/0000-0001-5126-299X
Pooja N
Poore A
Porter C https://orcid.org/0000-0002-6398-5519
Porteus C
Porto A https://orcid.org/0000-0002-9210-8750
Portugal S https://orcid.org/0000-0002-2438-2352
Potter T https://orcid.org/0000-0003-3201-6130
Potticary A https://orcid.org/0000-0002-1157-5315
Potts D
Potts J https://orcid.org/0000-0002-8564-2904
Potts M
Potvin D http://orcid.org/0000-0001-9296-9297
Powell M
Power E https://orcid.org/0000-0002-3064-2050
Prasad N https://orcid.org/0000-0002-0410-5518
Prescot O
Prescott C
Press C
Pressey R
Prestes-Carneiro LE
Preston J
Price C
Price E https://orcid.org/0000-0001-6042-7020
Prideaux G
Priede I https://orcid.org/0000-0002-5064-9751
Pringle A
Prior K
Privman E
Prochazka P https://orcid.org/0000-0001-9385-4547
Profico A
Prondvai E https://orcid.org/0000-0002-1284-8311
Prunier J https://orcid.org/0000-0003-4110-2567
Pull C
Puniamoorthy N https://orcid.org/0000-0001-9962-2640
Puts D
Püttker T
Qian F https://orcid.org/0000-0001-9656-1168
Qie L
Quintero J
Rabosky D https://orcid.org/0000-0002-7499-8251
Racicot R
Rádai Z
Raeymaekers J
Raine N https://orcid.org/0000-0001-6343-2829
Rajan R https://orcid.org/0000-0002-7770-3711
Rajčáni J
Rakoczy H
Ramalho M
Ramesh T
Ramos J
Ramsay C
Ranc N
Randy K
Rao A
Rasanen K
Rasmussen C
Rasplus J-Y
Ratcliff W
Ratikainen I https://orcid.org/0000-0001-9935-7998
Rayor L
Rebecca S
Rebolleda-Gomez M https://orcid.org/0000-0002-3592-4479
Reddon A https://orcid.org/0000-0002-3193-0388
Redmond A
Rees B https://orcid.org/0000-0001-5636-1700
Refai O
Regolin L https://orcid.org/0000-0001-8960-0309
Rehan S https://orcid.org/0000-0002-6441-5155
Reichert S
Reid C
Reif J
Reiser P
Reisinger R https://orcid.org/0000-0002-8933-6875
Reiss C
Reitman M
Rendell L https://orcid.org/0000-0002-1121-9142
Renoult J https://orcid.org/0000-0001-6690-0085
Resasco J https://orcid.org/0000-0003-1605-3038
Retallack G
Rey S https://orcid.org/0000-0002-3406-3291
Reynolds M
Rezende E
Ribas C
Rice A https://orcid.org/0000-0002-8598-9705
Richards R https://orcid.org/0000-0003-4131-2732
Richner H
Rider D https://orcid.org/0000-0002-1989-1873
Riebel K https://orcid.org/0000-0003-2373-8510
Riede T https://orcid.org/0000-0001-6875-0017
Riekkola L
Riesch R https://orcid.org/0000-0002-0223-1254
Riffell J https://orcid.org/0000-0002-7645-5779
Riginos C
Ringen E
Ringler E https://orcid.org/0000-0003-3273-6568
Ristevski J
Riutta T
Rivera M
Rivett D
Robert B
Robert K
Robert PA
Roberts M https://orcid.org/0000-0003-1688-5133
Roberts S https://orcid.org/0000-0003-3986-0254
Robertson D
Robeson M https://orcid.org/0000-0001-7119-6301
Robin M
Robin W
Robinson E https://orcid.org/0000-0003-1142-8347
Robinson L
Rocha L https://orcid.org/0000-0001-9046-8739
Roche B
Rockman M
Rodewald A
Rodrigues Simões T
Rodriguez R https://orcid.org/0000-0003-0262-0839
Rodriguez Santiago M
Rodriguez-Saona C
Roger R
Roitberg B
Rojas A
Roleda M https://orcid.org/0000-0003-0568-9081
Roman L https://orcid.org/0000-0003-3591-4905
Romanczuk P
Romeiras M
Romeis J
Römer H https://orcid.org/0000-0003-4326-0470
Ronco F
Rönkä K
Roopnarine P
Roos N
Rosa R
Rosales S
Rosas C
Rose E https://orcid.org/0000-0002-3750-0418
Rose P
Rosenheim J
Rosenkranz P
Rosenthal G
Ross C https://orcid.org/0000-0001-7764-761X
Rößler D https://orcid.org/0000-0003-4678-2231
Rosvall K https://orcid.org/0000-0003-3766-9624
Rouyer F
Rowan J https://orcid.org/0000-0002-5062-4044
Rowe M
Rowland H https://orcid.org/0000-0002-1040-555X
Roy H
Roy S https://orcid.org/0000-0001-7354-6459
Rozen D
Rubalcaba J
Rubin E
Rubolini D
Ruf T
Rugani R https://orcid.org/0000-0001-5294-6306
Ruiz-Aravena M https://orcid.org/0000-0001-8463-7858
Ruizhe L
Ruiz-Raya F https://orcid.org/0000-0002-4646-1975
Ruiz-Sainz J https://orcid.org/0000-0002-1447-1458
Ruiz-Trillo I https://orcid.org/0000-0001-6547-5304
Rumeu B
Rundle H https://orcid.org/0000-0002-8288-8888
Runemark A https://orcid.org/0000-0002-8976-5530
Russell A
Ruther J https://orcid.org/0000-0002-1295-347X
Ruuskanen S https://orcid.org/0000-0001-5582-9455
Ryan H
Ryan R
Ryan S https://orcid.org/0000-0002-4308-6321
Ryoya T
Sachell L
Sacristan S
Sadowska E https://orcid.org/0000-0003-1240-4814
Safran R
Sagarika P
Sahana K
Saito S
Sakata J https://orcid.org/0000-0003-2285-9310
Salas L
Salas-Gismondi R https://orcid.org/0000-0001-9990-8841
Salis A
Salisbury S
Sallan L
Saltzman W
Saluja S
Samuel F
San Millan A https://orcid.org/0000-0001-8544-0387
Sánchez-Tójar A https://orcid.org/0000-0002-2886-0649
Sandercock B
Sanders WJ
Sannolo M
Sansalone G
Santana S https://orcid.org/0000-0001-6463-3569
Santangelo G
Santos M
Santure A
Sarabipour S
Sarah W
Saramäki J
Sato Y
Satz H
Savage A
Sawicki G
Sayantani B
Sayer E
Sayol F https://orcid.org/0000-0003-3540-7487
Scanlan P https://orcid.org/0000-0002-6733-9653
Scanlon J
Scasta J
Schachat S https://orcid.org/0000-0003-3237-5619
Schaefer R
Schaerf T https://orcid.org/0000-0001-6642-8374
Schausberger P https://orcid.org/0000-0002-1529-3198
Scheele D
Scheiner S
Schellenberg G https://orcid.org/0000-0003-3681-6020
Scheuerl T https://orcid.org/0000-0001-5216-5630
Scheyer T https://orcid.org/0000-0002-6301-8983
Schielzeth H https://orcid.org/0000-0002-9124-2261
Schimmelpfennig R
Schineider Fachini T
Schlicht E
Schluter D https://orcid.org/0000-0003-1683-7836
Schmaljohann H https://orcid.org/0000-0002-0886-4319
Schmiedel U
Schmitt T
Schoepf V https://orcid.org/0000-0002-9467-1088
Schoonderwoerd R
Schoustra S https://orcid.org/0000-0001-7843-5539
Schrader M https://orcid.org/0000-0001-5432-6696
Schröder J
Schroeter E http://orcid.org/0000-0003-4314-2976
Schubert N https://orcid.org/0000-0001-7161-7882
Schülke O https://orcid.org/0000-0003-0028-9425
Schulte P https://orcid.org/0000-0002-5237-8836
Schulz-Kornas E https://orcid.org/0000-0003-1657-8256
Schuwerk T http://orcid.org/0000-0003-3720-7120
Schwander T
Schwaner MJ
Schwartz T https://orcid.org/0000-0002-7712-2810
Schweiger O
Schwertner C
Scopa C https://orcid.org/0000-0002-1519-9556
Scott G https://orcid.org/0000-0003-2328-8003
Seebacher F https://orcid.org/0000-0002-2281-9311
Segall M https://orcid.org/0000-0002-4913-2106
Segar S https://orcid.org/0000-0001-6621-9409
Seid M
Selbach C https://orcid.org/0000-0002-7777-6515
Sendova-Franks A https://orcid.org/0000-0001-9300-6986
Serena D
Seroussi E
Serra M
Servais T
Servin B
Seshadri A https://orcid.org/0000-0001-6128-3751
Sewell M
Seymour C
Shadwick R
Shafer A https://orcid.org/0000-0001-7652-225X
Shamble P https://orcid.org/0000-0003-4639-9339
Shaner P-J https://orcid.org/0000-0001-8112-4299
Shannon G https://orcid.org/0000-0002-5039-4904
Sharp A https://orcid.org/0000-0001-5117-5335
Sharples J https://orcid.org/0000-0002-7816-6989
Shaw J
Sheard C https://orcid.org/0000-0002-8259-1275
Shefferson R https://orcid.org/0000-0002-5234-3131
Shen S-F https://orcid.org/0000-0002-0631-6343
Sheppard P
Sherratt E https://orcid.org/0000-0003-2164-7877
Sherry D
Sherwood C https://orcid.org/0000-0001-6711-449X
Shikha K
Shima J https://orcid.org/0000-0001-5770-4859
Shimeld S https://orcid.org/0000-0003-0195-7536
Shocket M https://orcid.org/0000-0002-8995-4446
Shumway S
Shunichi T
Shykoff J
Sibert E https://orcid.org/0000-0003-0577-864X
Sibley R
Sigeman H https://orcid.org/0000-0002-1457-4174
Silcox M
Silk M
Silva B https://orcid.org/0000-0003-3877-1356
Silver R
Simon J-C
Simon S
Singh A
Siviter H https://orcid.org/0000-0003-1088-7701
Skeels A
Slade J http://orcid.org/0000-0002-4587-5033
Slate J https://orcid.org/0000-0003-3356-5123
Slavenko A https://orcid.org/0000-0002-3265-7715
Šlipogor V https://orcid.org/0000-0002-8842-4144
Slocombe K https://orcid.org/0000-0002-7310-1887
Sluys R
Smaers J
Smead R
Smiley T
Smiseth P https://orcid.org/0000-0001-6896-1332
Smit B
Smith C https://orcid.org/0000-0002-6470-3413
Smith O
Smith S
Smolla M https://orcid.org/0000-0001-6367-8765
Smulders T https://orcid.org/0000-0002-6673-7596
Sneddon L https://orcid.org/0000-0001-9787-3948
Snijders L https://orcid.org/0000-0003-0911-3418
Snowdon C
Sobel D
Sobral G
Socha J https://orcid.org/0000-0002-4465-1097
Soininen J
Sojung H
Sokolowski M http://orcid.org/0000-0002-7462-8007
Sol Balbuena M
Sol D https://orcid.org/0000-0001-6346-6949
Soler J https://orcid.org/0000-0003-2990-1489
Soma M https://orcid.org/0000-0002-8596-1956
Sommer R https://orcid.org/0000-0003-1503-7749
Sommer-Trembo C https://orcid.org/0000-0001-9952-2906
Sondhi Y https://orcid.org/0000-0002-7704-3944
Song F
Sørensen J
Sørensen M
Soulsbury C https://orcid.org/0000-0001-8808-5210
Southall B https://orcid.org/0000-0002-3863-2068
Spagopoulou F https://orcid.org/0000-0001-7876-5685
Spence J
Spivak M
Spocter M https://orcid.org/0000-0003-1174-7444
Spoelstra K https://orcid.org/0000-0001-8614-4387
Spyrou M
Sridhar H https://orcid.org/0000-0003-3286-0120
Srijan S
Srivastava D
St Clair S
Stach T
Staes N
Stagkourakis S
Stamenovic D
Stanback M
Stankowich T https://orcid.org/0000-0002-6579-7765
Stanley C
Stansbury A
Staples J
Staubach F https://orcid.org/0000-0002-8097-2349
Staudinger C https://orcid.org/0000-0003-0606-9407
Stayton T
Stein L https://orcid.org/0000-0002-4110-3562
Stein PK
Steinman A
Stephanie G
Stephens P https://orcid.org/0000-0003-1995-5715
Stern J https://orcid.org/0000-0001-8749-6392
Stewart A
Stewart T https://orcid.org/0000-0002-7965-5901
Stibor H
Stieglitz J
Stiels D
Stier A https://orcid.org/0000-0002-5445-5524
Stift M https://orcid.org/0000-0001-7801-9498
Stillman J
Stinchcombe J
Stireman J
Stockmaier S
Stone A https://orcid.org/0000-0001-8021-8314
Stothart M https://orcid.org/0000-0002-2863-908X
Strandburg-Peshkin A https://orcid.org/0000-0003-2985-6788
Strassmann JE
Strausfeld N
Street G https://orcid.org/0000-0002-1260-9214
Sturdy C
Sucena É http://orcid.org/0000-0001-8810-870X
Sudyka J
Sues H-D
Sueur C https://orcid.org/0000-0001-8206-2739
Suggitt A
Suh A https://orcid.org/0000-0002-8979-9992
Šulc M https://orcid.org/0000-0002-7866-1795
Summers K
Sundin J https://orcid.org/0000-0003-1853-4046
Suvi S
Suzuki T https://orcid.org/0000-0001-6405-7653
Svensson E https://orcid.org/0000-0001-9006-016X
Swanson D
Sweeny A https://orcid.org/0000-0003-4230-171X
Swei A
Swenson N https://orcid.org/0000-0003-3819-9767
Symonds M https://orcid.org/0000-0002-9785-6045
Szabo B https://orcid.org/0000-0002-3226-8621
Szathmary E https://orcid.org/0000-0001-5227-2997
Szeska C
Szöll?si G
Takahashi A https://orcid.org/0000-0002-8391-7417
Takahashi S
Takeshita F
Talas L https://orcid.org/0000-0001-5731-3890
Tamar R
Tams V
Tang JW
Tanimoto J
Tanya H
Tatarnic N https://orcid.org/0000-0001-8641-4412
Tate AT https://orcid.org/0000-0001-6601-0234
Tavera J http://orcid.org/0000-0003-4517-9238
Taylor D https://orcid.org/0000-0002-8559-7154
Taylor R https://orcid.org/0000-0003-3173-5432
Taylor T
Tchernichovski O
Tebbich S
Teerds K
Teets N https://orcid.org/0000-0003-0963-7457
Telemeco R https://orcid.org/0000-0002-0767-8565
Teletchea F
ten Bosch Q
Testo W
Testolin A https://orcid.org/0000-0001-7062-4861
Theraulaz G https://orcid.org/0000-0002-3062-4215
Theresa M
Theriault J
Thewissen JGMH
Thiago SF
Thierry B https://orcid.org/0000-0002-8065-093X
Thijs MPB
Thomas H
Thomas L
Thomas S
Thomas S
Thompson F https://orcid.org/0000-0001-7581-2204
Thompson G
Thompson Gonzalez N https://orcid.org/0000-0002-3195-1277
Thompson JC https://orcid.org/0000-0003-1627-4949
Thompson K https://orcid.org/0000-0002-1487-3254
Thompson N https://orcid.org/0000-0002-9273-3636
Thomsen M
Tibbetts E https://orcid.org/0000-0002-5625-892X
Tidau S https://orcid.org/0000-0003-0336-0450
Tierney A
Tierney M
Tigano A
Tihelka E https://orcid.org/0000-0002-5048-5355
Tilman A
Tilman D
Timi J
Timm L https://orcid.org/0000-0002-7285-0261
Tinghitella R https://orcid.org/0000-0002-0049-5539
Tingley M https://orcid.org/0000-0002-1477-2218
Tipton L https://orcid.org/0000-0002-5118-2671
Tissue DT
Tittensor DP
Togunov R https://orcid.org/0000-0001-9115-1207
Tökölyi J
Tomé C
Tomioka K
Tomiya S https://orcid.org/0000-0001-6038-899X
Tomlinson S https://orcid.org/0000-0003-0864-5391
Tooker J
Topper T
Törnroos A
Tosens T
Toth A https://orcid.org/0000-0003-0824-0933
Toups M
Towbin B
Townsend S https://orcid.org/0000-0003-1504-1801
Trabalon M
Tracey D
Trainor B
Tran N-H https://orcid.org/0000-0003-1668-2643
Trannoy S
Trevelline B https://orcid.org/0000-0002-4514-0467
Trillmich F https://orcid.org/0000-0003-4816-1156
Trillo P
Troscianko J https://orcid.org/0000-0001-9071-2594
Trumbo S https://orcid.org/0000-0002-4455-4211
Truskanov N https://orcid.org/0000-0001-7150-6568
Tseng J https://orcid.org/0000-0001-5335-4230
Tseng M
Tsuboi M https://orcid.org/0000-0002-0144-2893
Tuljapurkar S
Turner W https://orcid.org/0000-0002-0302-1646
Tyack P https://orcid.org/0000-0002-8409-4790
Tyler B
Uchiumi Y https://orcid.org/0000-0003-4449-1289
Udikovic N
Uhen M
Ujvari B https://orcid.org/0000-0003-2391-2988
Ulrich W
Urban M https://orcid.org/0000-0003-3962-4091
Usherwood J https://orcid.org/0000-0001-8794-4677
Uyeda J
Valadares L
Valle D https://orcid.org/0000-0002-9830-8876
Vallortigara G https://orcid.org/0000-0001-8192-9062
Van Belleghem S https://orcid.org/0000-0001-9399-1007
van Bergen E https://orcid.org/0000-0002-9648-9837
Van Cleve J
van Daalen S
van den Heuvel K
van der Bijl W https://orcid.org/0000-0002-7366-1868
van der Krogt M v
van der Zee M https://orcid.org/0000-0002-8728-8646
van Doorn S
Van Dyck H
van Grunsven RHA
van Heerwaarden B
van IJzendoorn M
van Moorsel S
van Oers K
van Rees C
van Veelen M https://orcid.org/0000-0002-8290-9212
Van Wassenbergh S https://orcid.org/0000-0001-5746-4621
van Wijk RE
Vanderelst D
Vannatta T
Vannette R https://orcid.org/0000-0002-0447-3468
Vardy T
Vargas M
Vasconcelos T
Vega Thurber R
Veilleux C https://orcid.org/0000-0003-4441-7521
Veldman J
Velez A
Veller C
Veltsos P
Venditti C https://orcid.org/0000-0002-6776-2355
Vermeij G https://orcid.org/0000-0002-6450-5687
Vernasco B https://orcid.org/0000-0002-5561-7273
Verzijden M
Vesk P
Vickruck J https://orcid.org/0000-0003-3453-6543
Victor A
Victor A
Victoria H
Villavicencio N
Vincent M
Vinn O
Vinther J https://orcid.org/0000-0002-3584-9616
Visscher P
Viswanathan A https://orcid.org/0000-0002-1605-0614
Vizentin-Bugoni J https://orcid.org/0000-0002-6343-3650
Voigt D https://orcid.org/0000-0003-2772-8504
Vojtech S
Vojtech T
von Rueden C https://orcid.org/0000-0002-3225-5791
Vonda C
Vonk J
Voordouw M https://orcid.org/0000-0001-9384-0436
Vorobyev M https://orcid.org/0000-0001-7615-5816
Vorontsova M
Vullioud P https://orcid.org/0000-0003-3603-2322
Waal E
Wada H
Wagner D
Wagner M
Wagner M
Wagner P
Wahlberg N
Wainwright P
Walker J
Wallace J
Walters E https://orcid.org/0000-0002-9414-5758
Waltham D
Waltham NJ
Wanelik K
Wang B
Wang X
Waples R https://orcid.org/0000-0003-3362-7590
Ward A
Ward R
Ware J https://orcid.org/0000-0002-4066-7681
Warnock R https://orcid.org/0000-0002-9151-4642
Warren P
Warton DI
Waters J https://orcid.org/0000-0002-1514-7916
Waterton J https://orcid.org/0000-0003-3177-7667
Watts P
Wcislo W
Weaver L
Weaver R
Webb B
Webb J
Webb T https://orcid.org/0000-0003-3183-8116
Webb W
Webber Q https://orcid.org/0000-0002-0434-9360
Weber J
Weihrauch D https://orcid.org/0000-0002-3218-9093
Weiner J
Weinstein SB
Weis V
Weisbecker V
Weiss A https://orcid.org/0000-0003-0835-4906
Weissing F https://orcid.org/0000-0003-3281-663X
Weithoff G
Weitz J https://orcid.org/0000-0002-3433-8312
Weizhao S
Wells K https://orcid.org/0000-0003-0377-2463
Werner A
Werner C
West K
West-Eberhard M-J
Whalen J https://orcid.org/0000-0001-8774-0594
While G
Whitaker B
Whitehorn P
Whitehouse J https://orcid.org/0000-0003-2607-5492
Whiting J
Whitney D
Whitney NM
Whoriskey F
Wiberg A https://orcid.org/0000-0002-8074-8670
Wienecke B
Wiener M http://orcid.org/0000-0001-5963-5439
Wierzbowska I
Wigby S https://orcid.org/0000-0002-2260-2948
Wiggins J
Wikenros C https://orcid.org/0000-0002-2825-8834
Wild G https://orcid.org/0000-0001-7821-7304
Wilfert L
Will R
Wille M https://orcid.org/0000-0002-5629-0196
Willems R
Williams J
Williams S https://orcid.org/0000-0002-3751-7703
Williams T https://orcid.org/0000-0002-6416-9441
Wilson C
Wilson J
Wilson L
Wilson M https://orcid.org/0000-0003-3073-4518
Wilson R http://orcid.org/0000-0001-7740-7771
Winberg S
Winker K
Winternitz J
Winters S https://orcid.org/0000-0002-9561-5950
Witt C https://orcid.org/0000-0003-2781-1543
Witte K
Wittig R https://orcid.org/0000-0001-6490-4031
Wittman T https://orcid.org/0000-0002-8679-4782
Witzel C https://orcid.org/0000-0002-1663-7618
Wolfe J
Wolff G
Wolfner M
Wolsan M https://orcid.org/0000-0002-2083-7743
Womack T
Wong M https://orcid.org/0000-0001-6393-6453
Wong RY https://orcid.org/0000-0003-0236-672X
Woodgate J
Wootton K
Wray G
Wright D
Wroe S https://orcid.org/0000-0002-6365-5915
Wu A https://orcid.org/0000-0002-1801-5716
Wu C
Wu N
Wunder M
Wurm Y https://orcid.org/0000-0002-3140-2809
Wynne C
Ximenes N
Xu S
Yamamichi M https://orcid.org/0000-0003-2136-3399
Yamashita N
Yampolsky L
Yang J
Yang L
Yang L
Yang Y
Yang Z
Yen-Wen W
Ying D
Yining C
Yip E https://orcid.org/0000-0002-3543-3717
Yo Y
Young H https://orcid.org/0000-0003-0449-8582
Yovel G https://orcid.org/0000-0003-0971-2357
Yseult H-B
Yukilevich R
Yusuf A https://orcid.org/0000-0002-8625-6490
Zack S
Zaffaroni Caorsi V
Žagar A
Zahra K
Zajitschek S https://orcid.org/0000-0003-4676-9950
Zamora S https://orcid.org/0000-0002-3917-4628
Zardoya R https://orcid.org/0000-0001-6212-9502
Zattara E https://orcid.org/0000-0002-9947-9036
Zavorka L https://orcid.org/0000-0002-0489-3681
Zefferman M
Zegni T
Zeng H https://orcid.org/0000-0002-5728-7896
Zhan R
Zhang H https://orcid.org/0000-0002-2674-3035
Zhang J
Zhao L
Zhao Q
Zhaoping L
Zhou X https://orcid.org/0000-0002-2385-8224
Zietsch BP https://orcid.org/0000-0003-0274-6140
Zinner D https://orcid.org/0000-0003-3967-8014
Zwolak R https://orcid.org/0000-0002-7665-5033

